# 3D genomics and its applications in precision medicine

**DOI:** 10.1186/s11658-023-00428-x

**Published:** 2023-03-06

**Authors:** Mengjie Chen, Xingyu Liu, Qingyou Liu, Deshun Shi, Hui Li

**Affiliations:** 1 State Key Laboratory for Conservation and Utilization of Subtropical Agro-Bioresources, College of Animal Science and Technology, Guangxi University, Nanning, 530004 Guangxi Province China; 2 Guangdong Provincial Key Laboratory of Animal Molecular Design and Precise Breeding, School of Life Science and Engineering, Foshan University, Foshan, 528225 China

**Keywords:** 3D genomics, Chromatin conformation, Gene expression regulation, Hi-C, Precision biology, Precision medicine

## Abstract

Three-dimensional (3D) genomics is an emerging discipline that studies the three-dimensional structure of chromatin and the three-dimensional and functions of genomes. It mainly studies the three-dimensional conformation and functional regulation of intranuclear genomes, such as DNA replication, DNA recombination, genome folding, gene expression regulation, transcription factor regulation mechanism, and the maintenance of three-dimensional conformation of genomes. Self-chromosomal conformation capture (3C) technology has been developed, and 3D genomics and related fields have developed rapidly. In addition, chromatin interaction analysis techniques developed by 3C technologies, such as paired-end tag sequencing (ChIA-PET) and whole-genome chromosome conformation capture (Hi-C), enable scientists to further study the relationship between chromatin conformation and gene regulation in different species. Thus, the spatial conformation of plant, animal, and microbial genomes, transcriptional regulation mechanisms, interaction patterns of chromosomes, and the formation mechanism of spatiotemporal specificity of genomes are revealed. With the help of new experimental technologies, the identification of key genes and signal pathways related to life activities and diseases is sustaining the rapid development of life science, agriculture, and medicine. In this paper, the concept and development of 3D genomics and its application in agricultural science, life science, and medicine are introduced, which provides a theoretical basis for the study of biological life processes.

## Introduction

DNA in the nucleus is highly folded and condensed to form chromatin with a specific three-dimensional advanced structure, rather than a single linear structure. This form of existence ensures that DNA remains stable as genetic material. The purpose of genomics is to collectively characterize and quantify all the genes of an organism, and to study their interrelationships and effects on the organism [1]. Genomics mainly studies the structure, function, evolution, localization, and editing of genomes, as well as their effects on organisms. Genomics research has been developing for more than 30 years since the start of the Human Genome Project [2]. In 1990 and 2003, scientists came together to launch the Human Genome Project (HGP) and Encyclopedia of DNA Elements (ENCODE) [3], which respectively define the important genes and linear structure of the human genome and parse all the functional components in the human genome. A series of new and different types of DNA and RNA are revealed and interpreted, including proteins involved in the process of transcription, translation, and other biology, enabling collaboration and information transfer. Therefore, scientists are faced with the new challenge of achieving precise and spatiotemporal individual functions of cells/organisms through different expression regulation modes. Since then, the focus of genomics research has been genome annotation to explore different genes at the genome level as well as the molecular mechanism of labor division and cooperation of each level, namely three-dimensional space structure in different genes, the coding of the interaction between the transcriptional regulatory elements, and the function of genes in a particular cell/biological individual expression regulation of biological effect. These research areas are part of the field of three-dimensional genomics.

Research on the genome has gradually been deepening, starting from the one-dimensional (gene sequence) and two-dimensional (interaction of different sequences) levels and gradually deepening to the three-dimensional (spatial conformation of chromatin) and four-dimensional (variation of sequence over time) levels, which has greatly promoted the development of genomics. Three-dimensional genomics is an emerging discipline that studies precise transcriptional regulation from the whole genome level. It aims to analyze the three-dimensional structure of genomic DNA in organisms, map individual transcriptional interaction networks of organisms, and explore the interaction between regulatory elements and genes, as well as to clarify the specific molecular mechanism of gene expression in time and space to achieve precise capture of gene regulation.

In 2002, with the chromatin conformation capture (3C) [4–6] technology coming out, the three-dimensional genome related research entered a new era, after which circular chromatin conformation capture technology (circular chromatin conformation capture technology that can be sequenced) was released after another 4C [5, 6] and chromatin conformation capture carbon copy technology (5C) [5, 7] was applied to multipoint interaction detection. These successively opened the door to the three-dimensional structure of chromatin. In 2015, a global cooperative project-the “4D Nuclear Bodies Project” was launched, and the research of 3D genomics began to enter an era of rapid development. Scientists will be in a few years, or even longer, from space (3D) and time (four-dimensional) perspective study to study the formation principle of cell nucleus structure and explore the influence of cell nucleus on gene expression, cell function, development, and disease occurrence. To have a more in-depth understanding of the popular subject of 3D genomics, this article briefly reviews the research progress of 3D genomics, including the development of 3D genomics technology, the characteristics of 3D genomics technology, and the application of 3D genomics in different fields, to provide strong research evidence and new ideas for the further research of three-dimensional genetics, provide theoretical knowledge for precision biomedicine, and promote the development of human precision medicine.

### The birth of 3D genomics and its main technologies

Unlike traditional biology, where the research objects range from “prokaryotes to eukaryotes,” the initial research objects of three-dimensional genomics are mice and humans. The completion of the HGP project in 2002 and the Encyclopedia of DNA Elements Project (ENCODE) [3] project in 2011 allowed scientists to interpret and annotate the characteristics and functions of different sequences in the genome, including coding sequences, noncoding sequences, transcripts, noncoding RNAs, microRNAs, transcription regulatory elements (enhancers, insulators, silencers), transcription factor binding sites, viscous protein binding sites, etc. [3]. At the same time, scientists realized that the spatial structure of the genome is not linearly arranged on the chromosomes. Its 3D structure is important for DNA replication, gene transcription regulation, chromatin condensation and separation, etc. [8]. At the same time (2002), the invention of 3C technology made it possible to use molecular biology methods to explore whether there is an interaction between two target genes, which also laid a solid theoretical foundation for the generation of three-dimensional genomics [9, 10]. In 2009, ChIA-PET [11] and Hi-C [12] technologies were invented. These two technologies allow scientists to elucidate the interactions between different genes and transcriptional regulatory elements at the whole genome level. This has become a milestone in the development of 3D genomics, and it also marks the coming of the era of 3D genomics.

**Fig. 1:**
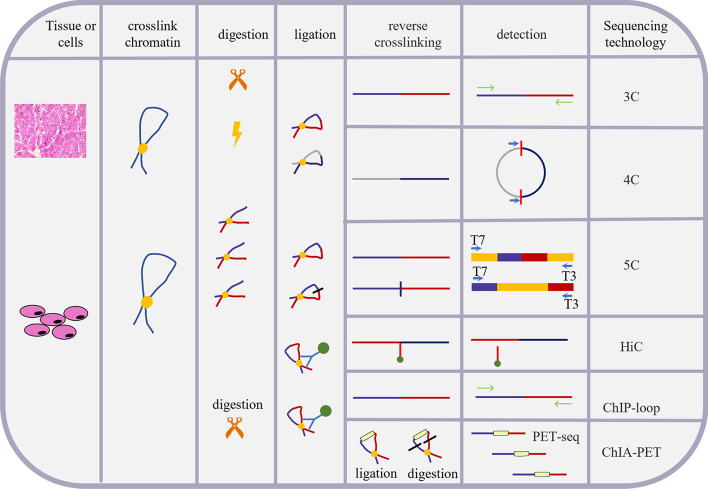
Overview of chromosome conformation capture technology (3C) and 3C-based technology. Formaldehyde fixation is cross-linked, followed by digestion and ligation with specific enzymes. Subsequently, the ligation products are enriched and the contact frequency is determined

**Fig. 2:**
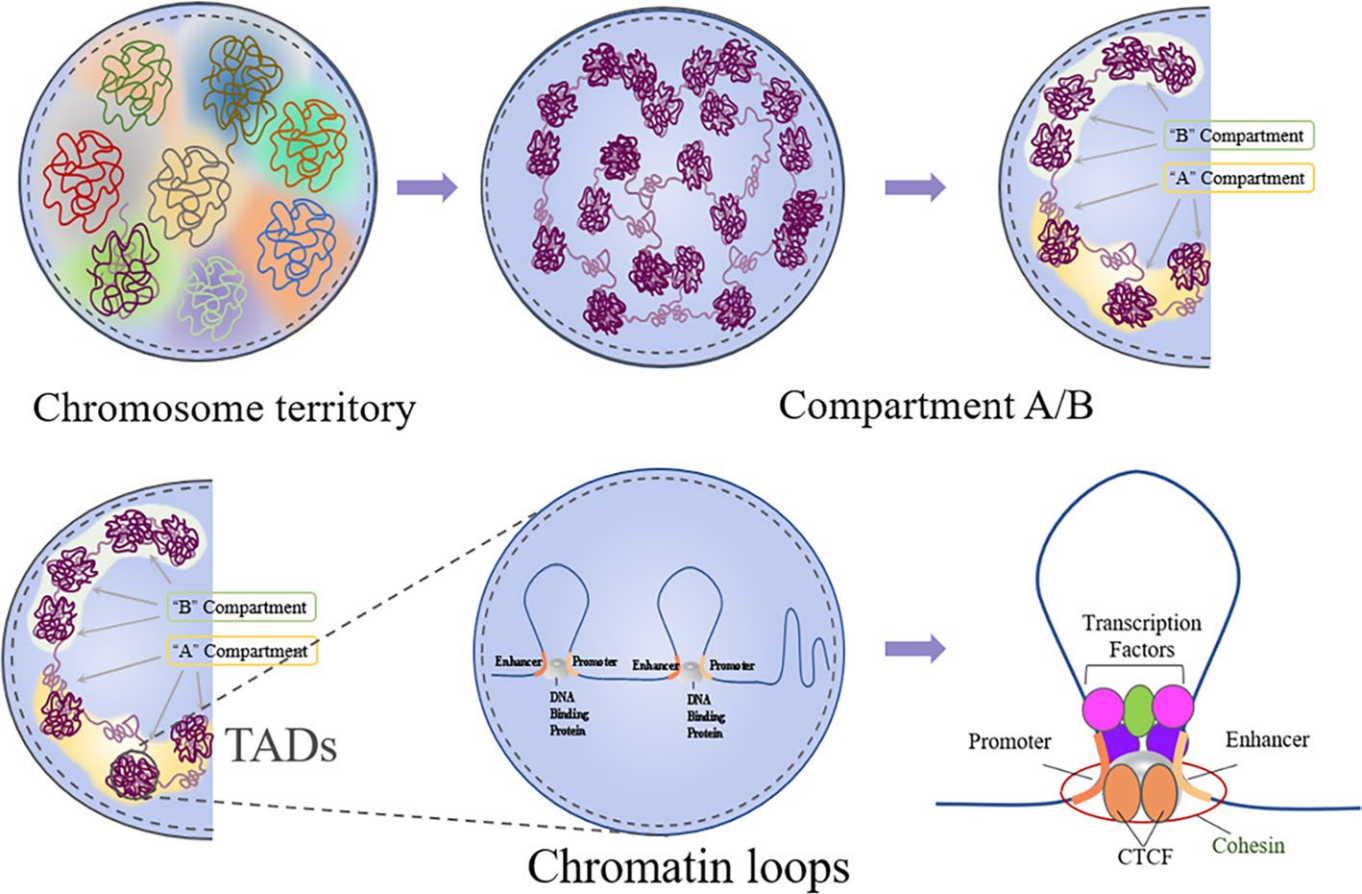
The 3D genome’s hierarchical organization. Chromosome territory refers to the region of the nucleus occupied by chromosomes. The entire genome can be partitioned into two multi-MB spatial sections, which can be called “A” and “B” compartments. There are frequent intracompartmental interactions, while there are fewer intracompartmental interactions. “A” compartments: open chromatin that is highly expressed and gene-rich, and has a high GC content, as well as histone indicators for active transcription, and is frequently found in the nucleus’s interior. “B” compartments: closed chromatin with inactive expression, gene deficit, and compact structure, carrying histone markers of gene silence, and located on the nucleus’s periphery. Topological domains are areas of the genome that interact with one another more frequently in chromatin compartments (TAD). These areas are conserved in various cells of different animals and are rich in CTCF and adhesive proteins, and CTCF, cohesive proteins, and mediators mediate the creation of enhancer promoter rings in these locations

In recent years, innovations in technologies and tools related to three-dimensional genomics research have emerged one after another (Fig. 1). 3C technology-based 4C [4], 5C [7], in situ Hi-C (2013) [13], hybridization probe Capture-Hi-C and CHi-C (2014) [14, 15], DNase Hi-C (2015) [16], DNase Capture-Hi-C [16], and in situ DNase Hi-C (2016) [17] and single-cell Hi-C technology [18] and BL-Hi-C [19] have been invented. These techniques use proximal junctions to detect chromatin interactions between spatially close DNA sequences, which has been applied to analyze the 3D genome structures of various cell types and model organisms, and have found that genomes are generally organized into various hierarchical structural units, including A/B compartments and subcompartments, topological correlation domains (TADs) [20], and chromatin interaction loops [21]. These series of research results show that, in the nucleus, DNA containing one or more genes and regulatory elements can be separated spatially to form topologically associated domains (TAD) [20]. The open or closed areas of chromatin can alternately form functionally related A/B compartments [12, 21]. Due to random collisions, two linearly distant gene loci will recognize the remote regulatory elements at a higher frequency, so as to realize the loop spatial conformation of long-distance transcription regulation, and then form a complex network structure [21, 22]. Therefore, chromatin is a specific spatial conformation formed by orderly folding in a hierarchical form in the nucleus (Fig. 2). [23–25]. Chemical crosslinking has assisted in adjacent junction capture technology (CAP-C) [26], DNA adenine methyltransferase (Dam)C [25], etc.

Different technologies such as ChIP-Llop, PLAC-seq [22], HiChIP [27], and long-read ChIA-PET [28] based on ChIA-PET [11] technology have also been successfully applied to related research fields. Through remote interactions mediated by specific transcription factors/genes, these studies have discovered a large number of interactions between different transcriptional regulatory elements, enhancers, and genes that determine the identity and status of cells, thereby constructing an intracellular transcriptional regulatory network [29–33] (Table 1). In addition, genome-wide RNA–protein interaction capture technologies have also been developed, such as RIP-seq [34] and CLIP series technologies, including HITS-CLIP, PAR-CLIP, iCLIP, CRAC, etc. [35–37]. Recently, the genome-wide DNA–RNA interaction capture technology GRID-seq [38] has also been developed. This technology can provide the most detailed promoter–enhancer-regulated RNA three-dimensional interaction map of the nuclear genome that is closest to the true transcription situation. This technology provides the possibility to explore the DNA–RNA of the whole genome. CRISPER–Cas9-based multicolor fluorescent labeling [39] and in situ biotin-labeled dCas9 technology (CAPTURE) [40] have also been applied in three-dimensional genome research.

**Table 1:** Characteristics and application of 3D genomics technology

Assay	Full assay name	Theory	Application	Features (interactions between DNA sequences)	References
3C	Chromosome conformation capture	Formaldehyde crosslinking, fragment DNA, adjacent ligation, DNA purification, forward PCR	The C-series, which is the basis of all C technologies, identifies known interactions between DNA with minimal throughput, whereas 3C can verify only one interaction	One to one	[5, 134]
4C	Chromosome conformation capture-on-chip	Formaldehyde crosslinking, fragmented DNA, nearby connection, DNA purification, reverse PCR, ChiP analysis or sequencing by adding adapter	Identification of a known interesting fragment and the interaction of multiple genes, high throughput, but there is a random positive connection caused by false positive	One to many	[5, 135]
5C	Chromosome conformation capture carbon copy	Formaldehyde crosslinking, fragment DNA, universal connector linking, adjacent linking, DNA purification, forward PCR	All interactions in a region can be detected, but that region is generally < 1 Mb. Coverage issues also make the technique unsuitable for whole genome sequencing	Many to many	[5, 7]
ChIA-PET	Chromatin interaction analysis by paired-end tag	Formaldehyde/EGS crosslinking, cell lysis, ChIP, linker nearby connection, tagmentation, PCR, sequencing	Capturing genome-wide interaction of target proteins in the nucleus. Compared with Hi-C, which has the advantage of high resolution, it is possible to reconstruct the genome three-dimensional structure together with Hi-C	Many to many + protein specific	[28, 136]
Capture-C	Chromosome conformation capture coupled with oligonucleotide capture technology	Formaldehyde crosslinking, Fragmented DNA, Nearby connection, Purified circular DNA, Probe hybridization, Sequencing	By hybridization of biotin DNA probes, identification or searching for multiple purposes of known multiple segments intercropping, enrichment sequencing to improve fluence, but there is a random positive connection caused by false positive	Many to all	[15]
Capture-HiC	Hi-C coupled with oligonucleotide capture technology	Specific probe labeling, Formaldehyde crosslinking, Enzymes fragment DNA, End biotin labeling, Dilute the solution, Nearby connection, Purified circular DNA, Sonication, Immunomagnetic beads precipitation, Sequencing	Specific probes were used to study only the target region to capture chromatin interactions in the region where the probe was located (e.g. the promoter region)	Many to all	[66]
PLAC-seq	Proximity ligation-assisted ChIP-seq	Formaldehyde crosslinking, fragment DNA, biotin labeling, ligating, cell lysis, ultrasound, ChIP, DNA purification, immunomagnetic bead precipitation, sequencing	Capture genome-wide interactions of target proteins in the nucleus	Many versus all + antibody to recapture	[137]
HiChIP	Protein-centric chromatin conformation assay	Formaldehyde crosslinking, fragment DNA, biotin labeling, ligating, cell lysis, ultrasound, ChIP, DNA purification, immunomagnetic bead precipitation, sequencing	Capture genome-wide interactions of target proteins in the nucleus	Many versus all + antibody to recapture	[27]
Hi-C	High throughput chromosome conformation capture	Formaldehyde crosslinking, enzymes fragment DNA, end biotin labeling, dilute the solution, nearby connection, purified circular DNA, sonication, immunomagnetic beads precipitation, sequencing	Capturing genome-wide interactions within the nucleus, the resolution is low at present, and there are random connections and background noise	All to all	[12, 138]
In situ Hi-C	In situ chromosome conformation capture	Formaldehyde crosslinking, enzymes fragment DNA, end biotin labeling, dilute the solution, nearby connection, purified circular DNA, sonication, immunomagnetic bead precipitation, sequencing	Capture genome-wide interactions of target proteins in the nucleus	All to all	[13]
ATAC-seq	Assay for transposase-accessible chromatin with high-throughput sequencing	Nucleoplasmic separation, chromatin fragmentation, joint labeling, DNA purification, PCR amplification, double-terminal sequencing	Transposase splices open sections of nuclear chromatin in a specific spatiotemporal context to access the regulatory sequences of all active transcription in the genome	Genome-wide	[139]
ATAC-see	Assay of transposase-accessible chromatin with visualization	Nucleoplasmic separation, chromatin fragmentation, fluorescence labeled splice, DNA purification, PCR amplification, double-terminal sequencing	Transposase can obtain the regulatory sequences of all active transcription in the genome in a specific space and time by slicing open chromatin regions in a specific space and time, and three-dimensional immobile nuclei can be visualized by slicing open chromatin regions in a specific space and time	Genome-wide	[140]
Single-cell ATAC-seq	Single cell assay for transposase accessible chromatin with high-throughput sequencing	Nucleoplasmic separation, chromatin fragmentation, joint labeling, DNA purification, PCR amplification, double-terminal sequencing	Transposase splice open portions of nuclear chromatin in a specific temporal and spatial context to obtain regulatory sequences of all active transcription in the genome within a single cell	Genome-wide	[18, 141, 142]

Current innovations in microscopy technology, such as the combined method ChromEMT [41], ensure its position in 3D genomics research. With the successful development of optical microscopes with a resolution of 30 nm, scientists invented a new technology-ChromEMT, by combining chromatin dyes with electron microscopy tomography [42]. These techniques were the first to observe chromatin structure directly in the nucleus through electron microscopy. It was found that chromatin existed in disordered curved chains with a diameter of 5–24 nm at any stage of the cell cycle, but the concentration distribution in three-dimensional space varied with the cell stage. This new discovery “upends” the classical model of chromatin multihierarchy [43]. It is foreseeable that these studies will greatly promote the smooth implementation of the “4D nucleosome project” and will also greatly enhance scientists’ understanding of the relationship between genome-wide spatial conformation and gene transcription regulation [44–46]. Indeed, new techniques have been developed to resolve single-cell maps of three-dimensional genomes while preserving information about the spatial location of these cells in their tissue environment, known as the emerging “spatial genomics.” For example, optical reconstruction of chromatin architecture (ORCA) and Hi-M techniques have been applied to *Drosophila* tissues [47, 48], while multiplexed imaging of nucleome architectures (MINA) is the first method to perform chromatin tracing in mammalian tissues [49], revealing the presence of genomic regions associated with nucleosomal and chromatin markers in different cellular genomes. Moreover, some of these chromatin-associated regions appear to be cell-type specific, while others, primarily nuclear speckle-associated regions, are more conserved across cell types.

Multiplexed error-robust fluorescence in situ hybridization (MERFISH) is applied for high-throughput in situ recognition of thousands of RNA molecules in cells [50], and has been applied to in situ imaging of genomic DNA [51]. Using MERFISH to image TAD structures with ultrahigh-resolution microscopy (STORM) requires further specificity and sensitivity. Secondary hybridization enables MERFISH to trace chromatin structure at super-resolution, which compensates for the defect of the single-cell Hi-C (scHi-C) method’s inability to clearly see the structure of TAD at the single-cell level [52].

To further explore the detailed chromatin structure, researchers have made full use of the fluorescence in situ hybridization (FISH) technique, including exposure to enhancer promoters, but this method is limited to fixed cells and is incapable of elucidating the spatiotemporal genome dynamics. The emergence of genome editing technologies, such as clustered regularly interspaced short palindromic repeats (CRISPR), has revolutionized various techniques in biomedical research, including imaging tools for chromatin interaction to target specific genomic locations of interest in living cells [53, 54]. For example, CRISPR was introduced in living cells by labeling the enhanced green fluorescent protein (EGFP) to the endonuclease-deficient Cas9 (dCas9) protein, which, in combination with imaging techniques, was recruited specifically by small guide RNA (sg) [55]. The method has been applied to other derivatives of the dCas9 linear homolog using different-colored fluorescent protein tags, facilitating the visualization of multiple loci and the distances between them [56, 57]. In addition to dCas9, sgRNA is modified by fusion scaffolds with fluorescently labeled viral RNA recruiting viral proteins, allowing simultaneous multicolor imaging of multiple genomic sites [57–59]. These methods are efficient when targeting repetitive sequences in the genome by easily amplifying fluorescent signals, but are not applicable to targeting nonrepetitive genomic regions where signals cannot be strongly amplified for detection. CRISPR-tag and chimeric array of gRNA oligonucleotides (CARGO) have been developed to enhance the signal of dCas9–sgRNA complexes at the nonrepetitive genomic sites [54]. CRISPR-tags are DNA tags that assemble together repeats of two to six CRISPR targets from *Cryptococcus* that have been knocked into specific human protein-coding genes and recruit dCas9-GFP proteins for imaging [60]. CARGO is capable of delivering 12 guide RNAs cloned in a single plasmid into a single cell, and has been applied to target 2 kb regions of CREs, revealing its dynamics in real-time embryonic stem cell (ESC) [61].

## Three-dimensional structure of genome chromatin

Accurate gene expression is a prerequisite for cells and individuals to maintain normal life activities, and the three-dimensional structure of genomic chromatin is the structural basis for precise gene expression regulation. DNA molecules about 2 m in length in mammalian cells are stored in the nucleus with a diameter of about 8 µm in a highly folded and condensed chromatin form, forming a complex and orderly three-dimensional structure, allowing gene expression far away on the linear genome. Regulatory elements can be sufficiently close to their target genes in three-dimensional space, so as to play a fine regulatory role as functional elements. Studies have shown that changes in the three-dimensional structure of genomic chromatin can lead to abnormal gene expression and its regulatory mode, which in turn causes phenotypic changes. Three-dimensional genomics is a research hotspot in the post-genomics era and the post-genome-wide association study (GWAS) era. Compared with the study of two-dimensional linear genome functions, three-dimensional genomics can analyze the precise regulation mechanism of functional elements on gene transcription by exploring the three-dimensional structure of genome chromatin and its regulation of gene expression. Up to now, the three-dimensional structure of chromatin has been resolved in many species, including humans, mice, fruit flies, chickens, parasites, rice, cotton, and corn. In addition, Functional Annotation of Animal Genomes (FAANG) has initially completed the three-dimensional genome study of liver tissues of pigs, chickens, and goats. Due to factors such as DNA sequence, chromosome morphology, number, and tissue specificity of functional elements, the three-dimensional structure of chromatin must be different in the same tissue of different species or in different tissues of the same species. The three-dimensional structure of genomic chromatin is the structural basis for precise gene expression regulation. Bickmore et al. found that the nucleus during the interphase of chromosome division was analyzed and found that there are longer fragments of nuclear chromatin interval and chromosome territory (chromosome territory, CT) in the nucleus, and there are also enhancer–promoter junction regions. These chromatins are three-dimensional. Structure has an important influence on cell gene expression and regulation. With the help of Hi-C and other technologies, researchers have discovered the existence of topologically associated domains (TADs). TADs, as the basic unit of genome folding, exist stably in cells of various species and influence gene expression to a certain degree. The TADs in the nucleus exist in a relatively large structural unit, the chromatin compartment. The chromatin compartment is a manifestation of the apparent state of the genome and is closely related to chromatin activity. Inside TADs, there is a finer folding structure called the chromatin loop (CL), which is usually formed by the interaction of promoters and enhancers (distal) and is the basic function of directly regulating gene expression.

### Chromosome territory

Using microscopic observation technology and chromatin conformation capture technology, it is found that each chromosome tends to occupy non-overlapping nuclear regions independently. These regions are called chromosome territories [62, 63]. Within the territory of chromosomes, the genome is not randomly distributed but is related to gene transcription activity. Gene enrichment regions tend to be distributed at the borders of chromosome boundaries, although this is not a common phenomenon. For example, Hoxd gene is activated and expressed in mouse e9.5 embryonic limb buds, but the gene is not transferred to the border of the chromosome when activated [64, 65]. There are also interactions between different chromosome territories, especially at the borders of chromosome territories.

### Chromatin compartment A/B

At the Mb level, genomic regions with similar chromosomal characteristics have obvious interactions. For example, there are interactions between the transcriptional activation regions of the genome. These regions usually have higher gene density, chromatin openness, and histone modifications [12]. On the contrary, transcription repression regions, usually gene deserts and heterochromatin regions, will interact with other transcription repression regions. This chromosomal region at the Mb level is called a chromosome compartment, where compartment A is a transcriptional activation area, and compartment B is a transcriptional repression area [12, 66]. This structure was confirmed by Hi-C and microscopic observation techniques [67, 68]. The spatial distribution of chromatin is usually related to various nuclear structures. For example, compartment A is often found in the interior nuclear space, while compartment B is usually located in the nuclear lamina and nucleoli. In human fibroblasts, approximately 40% of the genome is associated with laminin [46].

During the differentiation of mouse embryonic stem cells (mESCs) into neural progenitor cells and further into astrocytes, the interaction pattern between hundreds of gene loci and nuclear lamina gradually changed. The spatial separation of chromatin is not limited to intrachromosomal compartments, but also applies to different interchromosomal compartments [69]. A recent study identified the interaction of compartments between chromosomes. This study found that the interchromosomal transcription-repressive regions are assembled at the nucleolus and usually contain centromeres and ribosomal DNA regions. The finding is consistent with the previously observed nucleolar-related chromosomal domains. On the contrary, the transcription activation region usually assembles at the nuclear speckles. Mechanistically, the location of the B-compartment domain at the periphery of the nucleus depends on the laminin B receptor, laminin A, and laminin C, because the loss of all three proteins will cause the heterochromatin to relocate to the inside of the nucleus [13].

### Topological associated domain (TAD)

Another structural level of three-dimensional chromatin is TAD [70, 71]. TAD was originally identified by Hi-C and 5C [72]. In the 2D interaction heat map, it appears as an interaction box on the diagonal. TAD is separated from adjacent regions by a clear boundary, forming an independent regulatory unit, and its main function is to limit the interaction distance of regulatory elements. TAD boundaries are usually highly conservative, but there are also some cell-specific TAD boundaries. TAD borders usually have a large number of chromatin structural proteins CTCF and adhesion proteins (TAD borders in plants generally lack insulating proteins and the borders are not obvious), which play an important role in maintaining the structure and stability of TAD [46, 73, 74]. The TAD boundary not only guides the folding of chromatin into high-level structures, but also correctly guides the long-distance transcriptional regulation. Changes in this boundary can lead to disordered gene regulation. TAD borders usually have histone modifications related to gene activation, such as H3K4me3 and H3K36me3. As the depth of Hi-C sequencing increases [33, 75], using the latest algorithm (Arrowhead algorithm), at a resolution of 5 kb, it is found that the three-dimensional structure of chromatin can be divided into compartmental domains (compartmental domains) and CTCF (CCCTC-binding factor) loops. The formation of the compartment domain has nothing to do with CTCF; it is caused by chromatin state and gene transcription, and the CTCF loop is formed by adhesion protein and CTCF. The compartment area is smaller than TAD, and can be further divided into A/B compartment area [33, 67]. Thus, there may be different compartments in TAD.

### Chromatin loop

With the increase of the sequencing depth, more fine interaction peaks are further found in the TAD, which are remotely generated by the regulatory elements in the TAD, which are called interaction loops. Similar to the formation of a loop at the two ends of the TAD, the long-distance interaction between regulatory elements will also make the chromatin loop. Therefore, broadly speaking, the chromatin loop includes TAD and loop structures, and loop is usually hundreds of kilobases, which is much smaller than TAD. Loop is a hotspot in three-dimensional genomics research, which can effectively annotate the interaction of genome functional elements.

Since 2003, the human ENCODE project has revealed hundreds of thousands of genomic functional elements [3]. These regulatory elements play a vital role in the precise expression and regulation of genes. However, the early functional studies of two-dimensional linear genomes could not systematically provide the target gene information of these regulatory elements. Moreover, it is impossible to explain the mechanism through which these regulatory elements interact with target genes that are tens of thousands or even hundreds of thousands of nucleotides away from them. In fact, in the nucleus of eukaryotes, chromatin follows certain rules for complex and orderly three-dimensional folding to form a specific three-dimensional structure of chromatin. This makes the regulatory elements that are far away from the linear genome and their target genes sufficiently close in three-dimensional space, so as to play the fine regulatory role of the functional elements.

With the advancement of the ENCODE project, people realized that this kind of long-distance precise gene expression regulation is widespread in the mammalian genome and is a necessary condition for the normal growth and development of the body. For example, *MYC* promoter and *PVT1* promoter can competitively interact with four enhancers in *PVT1* gene. When the *PVT1* promoter region is mutated, the interaction between the enhancer and the MYC promoter in the three-dimensional space is enhanced, which promotes the occurrence of cancer [76, 77]. In addition, knocking out the distal enhancer of *Sox9* resulted in gender reversal in mice [78].

### Chromatin-loop extrusion model

Loop and TAD are both formed by long-distance loops of genomic chromatin, collectively referred to as chromatin loops. Chromatin looping is caused by loop extrusion. The formation of loop is conducive to the interaction of regulatory elements and can regulate gene expression. TAD is usually larger than loop and can limit the interaction of regulatory elements within a certain range. The discovery of TAD and loop is defined with the sequencing depth and the corresponding algorithm, so the results obtained by using different algorithms and resolutions will be different.

According to reports, 86% of chromatin loops are anchored by CTCF [13], and 86% of chromatin loops are anchored by adhesion proteins subunit RAD21 [46]. Adhesin complex can form a ring structure and can move on chromatin. Adhesin can recruit NIPBL and MAU2 proteins and release them from chromatin through WAPL protein. The translocation of adhesion proteins on chromatin requires ATP, because nonspecific inhibition of ATPase or specific mutation of the ATPase domain in the adhesion proteins complex will inhibit this translocation. Gene transcription is also conducive to promoting the translocation of adhesion proteins, which in turn promotes the formation of their circular structures. Mechanistically, CTCF and Adhesin make genomic DNA form TAD through the “chromatin-loop extrusion” model. In this model, adhesion proteins squeeze chromatin outwards until adhesion proteins meet the chromosome boundary formed by CTCF. In this way, several ring structures are formed inside the TAD, which can promote the interaction within the TAD. In the Hi-C interaction matrix, these chromatin loops appear as high-frequency interaction peaks. It is worth noting that the CTCF binding sites at the two boundaries of loop are usually reversed, and their motifs are face to face. Changing the direction of the CTCF motif will destroy the loop and TAD formation. These results strongly indicate that CTCF will promote the formation of loops. In addition, deleting loop extrusion factors, adhesion proteins, or assembly factors NIPBL will cause the widespread disappearance or reduction of TAD and loop. However, there is currently no direct evidence that adhesion proteins can squeeze the chromatin ring. In addition, a recent 4C study found that knocking out CTCF-related TAD boundaries did not affect the mode of local chromatin interaction. Although the study did not evaluate the three-dimensional structure of the whole genome (for example, using 5C or Hi-C for evaluation), in addition to CTCF and adhesion proteins, other factors may also contribute to the formation of TAD [70, 79].

Although TAD is widespread in many species, single-cell Hi-C [80] studies have shown that TAD between single cells is not completely the same. For example, single-cell Hi-C analysis in mice showed that TAD was indeed found in a single cell, but the TAD is different between different cells. After knocking out the adhesion protein, although the TAD boundary of CTCF and the adhesion protein has disappeared, the single cell still has a TAD-like structure [81]. It is not clear whether there is a fundamental difference between TAD and nondependent TAD.

## Features of 3D genomics

### Spatial structure and three-dimensional transcriptional regulation

Before the emergence of three-dimensional genomics, research methods related to the spatial structure of chromatin or gene interaction were mainly based on low-throughput biological or physical technologies, such as DNA-FISH [9], chromatin karyotype analysis [82], yeast two-hybrid [83], immunoprecipitation [41], protein profile analysis [84], etc. The common features of these technologies are high requirements for pre-preparation work, complicated operations, high background noise, and the inability to more directly obtain the interaction information between different genes [22]. Three-dimensional genomics technology gets rid of these limitations, and enables scientists to directly obtain the DNA sequence and interaction information of different genes and transcriptional regulatory elements with high throughput without prior work. Therefore, scientists can study the molecular mechanism of chromatin spatial conformation, three-dimensional structure of genome, and the regulation of transcription of different genes.

Hi-C technology reveals the interaction between different genes and transcriptional regulatory elements in the whole genome in a “full-to-all” model, and analyzes the interaction mode of all DNA elements in chromatin, which has become a typical representative of three-dimensional genomics technology [12]. The application of this technology has successfully defined chromosome territories and chromatin compartments (including active or inactive regions), and constructed a three-dimensional genome structure with a resolution of 1 Mb [12]. However, short-distance imaging of chromatin interactions remains a technical bottleneck. The key to solving this problem is how to gather more fluorescence into a very short target region. To address this problem, a new technique called Tn5-FISH (Tn5 translocation-based fluorescence in situ hybridization) with a resolution of up to 1 kb has been developed by the research group of Juntao Gao. With the help of Tn5 transposase, it cuts DNA into small fragments with the shortest 40 bp through the “cut and paste” mechanism, while adding splice DNA sequences with known sequences to two segments of the fragment, and constructing probe library by PCR amplification to mark chromatin in the nucleus [85]. On this basis, Bing Ren et al. defined the topological association domains (TADs) and their sizes, and further studied the cooperative relationship between this region and the structural protein factor CTCF (CCCTC-binding factor) [71]. At the same time, they conducted in-depth research on the relatively dynamic sub-TAD, and proposed the concept of chromatin loops, which are considered the smallest unit of gene regulation [5]. Recently, scientists have invented a new 3C method, TiLED-MCC, which comprehensively demonstrates the interaction law and chromatin structure of all regulatory elements in these gene regions of mouse embryonic stem cells [86].

The ChIA-PET technology identifies the functionally related interactions between target genes or transcription factors and different DNA elements in the whole genome, which helps to directly interpret the connections between different interactions of the same gene-specific temporal and spatial expression patterns [12]. As Zhang et al. reported in 2013, using ChIA-PET technology to identify specific antibodies for RNA polymerase II, the study revealed 40,000 long-range interacting enhancer-promoters in three different cell lines [29].

Through the ChIA-PET data mediated by RNA polymerase II and CTCF, the researchers successfully mapped the genome-wide interaction map centered on the promoter, constructed a genome-wide transcriptional regulatory network [70], successfully integrated systematic analysis of different genomics or gene function data with three-dimensional genomics data, and successfully revealed a series of molecular mechanisms related to cancer [11, 87, 88], blood diseases [89, 90], noncoding RNA function [91], sex determination [78], and body immunity [92, 93].

There is no doubt that these latest research findings have laid a solid foundation for the further study of the three-dimensional transcriptional regulatory network of eukaryotic genomes. In the future, three-dimensional genomics research will be more concentrated in the construction of gene three-dimensional network, the discovery of key transcription factors/regulatory elements, and the biological effects of gene interaction on cells/individuals.

### Integration of multiple omics data

The main content of three-dimensional genomics research is the spatial conformation of the genome. By studying the spatial structure relationship between different genes and transcriptional regulatory elements [94], the cooperative relationship between different genes and transcriptional regulatory elements can be revealed. In short, its main content is to reveal the relationship between different elements in the genome.

To accurately analyze the influence or biological effects of the relationship between different elements in the genome on the functions of different genes, it is necessary to make detailed annotations on the biological effects of different genes, transcriptional regulatory elements, or other biological macromolecules involved in the transcriptional regulation of specific genes. Through the biological process of dynamic time and space, the biological effects of the spatial structure, including the downstream RNA transcription and expression pattern and the level of protein translation regulation, are used for in-depth research. In the process of studying the spatial structure of the three-dimensional genome and its influence on the transcriptional regulation of different genes, it is necessary to integrate other different types of omics data to define and annotate different elements in the whole genome. Only by combining the biological processes of temporal and spatial dynamics can scientists accurately and deeply carry out the next step in the interpretation of the relationship between gene interaction and function [95]. Zhao et al. analyzed pig tissues with a combination of different techniques, including ChIP-seq, Hi-C, RNA-seq, and ATAC-seq research technical systems. In this study, 199 groups of data containing 12 kinds of epigenetic regulation were obtained from Meishan pig, Duroc pig, lean big white pig and lard pig, and the genome of specific regulatory elements in pig tissue was analyzed. These findings serve as a foundation for future research on functional genomics and trait expression regulatory mechanisms.

At present, data related to gene expression and gene annotation in the genome are often used in this type of analysis, such as RNA-seq data that reveal gene expression levels in the genome, and ChIP-seq data that identify different nucleosomal properties or transcription factor binding sites, and GWAS data indicating possible biological significance in the whole genome [96–98]. The integration of these studies with 3D genomics data has deepened our understanding of existing gene functions and modes of action. However, integrating multiple data is an enormous challenge. Ideally, the integration of relevant studies requires multi-omics data covering all biological levels, intermediate steps from DNA to protein, all developmental stages from stem cells to death, and all cell types from stem cells to functional cells. In fact, only a small fraction of the required data is generated, and more data will be available in the future. Spatial transcriptome, Hi-C data will contribute to a better understanding of gene transcription and its regulation. In addition, histological data are sensitive to sex, ethnic genetic background, and other variation, the effects of which are not well presented in most public databases. Data on ethnic diversity remain generally unavailable. In 2019, researchers found that about 78% of GWAS individuals were of European ancestry [99]. The diversity of brain histology data is even smaller. For example, in the current version of the Accelerating Medicines Partnership Parkinson's Disease (AMP-PD), less than 4% of participants were non-white/Caucasian [100]. This is a huge problem for research aimed at being more inclusive. Projects such as the African Descent Neuroscience Study promise to fill this gap. The integration of data relies on large samples provided by research laboratories, but the number of samples is still far less than the number of traits. Before integrating multi-omics, researchers must reduce the dimensionality. Related studies have shown that classification based on weighted gene co-expression network analysis (WGCNA) co-expression modules can better cope with differences between datasets than classification based on raw gene expression. Other dimensionality reduction techniques such as support vector machines (SVM), random forests (RF), and singular value decomposition (SVD) are also commonly used to reduce overfitting problems. Advanced deep learning methods such as variational autoencoders (VAE) also output low-dimensional potential representations of high-dimensional data. Multiple test information and saliency criteria are concomitant issues.

## Applications of 3D genomics

### Interpreting the function of genes

Before the emergence of three-dimensional genomics, the main methods of gene function research were gene knockout, gene knockdown, gene fusion, and association analysis of population traits. The progress of related work mainly depends on whether the changes in gene phenotype or biological traits can be observed. If no changes in the relevant traits are observed, the research work will be suspended or suspended. The objects of these research work focus on the gene itself, and the research on the upstream and downstream pathways of gene function is mainly based on the support of different experimental hypotheses or indirect evidence. For example, Chen et al. reported in 2008 that, by integrating the binding sites of 13 different transcription factors in the embryonic stem cell genome, they found a series of binding sites related to gene transcription regulation activity. This work provides a new way for gene function and genome annotation, and has received extensive attention from scientists. However, due to the lack of the interaction between these binding sites and the target gene, and the limitations of technical conditions, the results of this study have not been widely used in the study of gene function [101].

In three-dimensional genomics, the identification of the mode of interaction between transcription factors and regulatory elements will clear the way for similar research. For example, in human red blood cells, researchers have found that, in the absence of the *GATA1* gene, the β-globulin promoter region can interact with locus control regions (LCR) to enhance gene expression [102]. Further studies have shown that, through the interaction between LCR and γ-globulin, the expression level of γ-globulin in the fetal period can be increased to 85% of the total globulin level [103]. The above results indicate that gene interactions dominated by three-dimensional spatial structure can determine developmental pathways or fate [104]. By determining the interconnection between these binding sites and target genes, scientists can further study the on/off of transcriptional regulation mode and the high/low level of gene expression under dynamic spatiotemporal conditions.

### Screening of key transcription factors or genes

The ultimate goal of genome-wide spatial conformation research is to study gene functions in depth and provide guidance or research clues for subsequent experiments or analysis. As a carrier of cell/biological individual genetic function information, the study of gene function or transcriptional regulation mode has always been the focus of scientific researchers. Especially the research on the functions and dynamic transcription regulation modes of transcription factors/key genes that have a significant impact on the biological characteristics of cells/biological individuals.

After obtaining the gene interaction/cooperation information on the three-dimensional structure, the relevant data can be used to identify, classify, and evaluate the biological effects of different types of gene interaction/cooperation data. These works will help researchers to further study gene interaction/assistance. At the same time, these research results can be integrated into the three-dimensional structure of the whole genome again.

Researchers can use these data to better analyze and identify the biological effects of different gene interactions/cooperation in the whole genome, and it is also helpful to identify different transcription factors/key genes at specific developmental stages, so as to further explore gene functions. For example, Zhang et al. reported in 2013 that, through improved ChIA-PET technology, three-dimensional transcriptional regulatory networks were constructed in embryonic stem cells and neural stem cells, and these networks confirmed the hypothesis that organisms/cells may have transcription factories. At the same time, some transcription factors/key genes such as *Oct4*, *Sox2*, *Klf4*, and *c-Myc* were found to exist in this network, and their interacting genes and transcriptional regulatory elements have also been successfully verified [29].

In other different research fields, certain transcription factors/key genes have also attracted the attention of many scientists, but their genome-wide gene interaction/assistance relationship is still unclear, for example, *MyoD* and *Myf5* in the differentiation and development of muscle cells [105], *Sox2 *and *Sox* gene families in the development of nerve cells [106], *IFN* and *IL* gene families in immune cells [107], *Ras* and *p53* in tumor cells [108], and *Hox* gene families in the development process [109]. How do these genes interact with other transcription factors/genes/regulatory elements? Do they also have a genome-wide transcriptional regulatory network like mouse embryonic stem cells? What role do they play in the dynamic development of cells/biological entities? These issues are awaiting continued attention and in-depth research by scientists in their future work.

### Enhancers and super enhancers

Enhancers are noncoding DNA elements that regulate gene function and transcription regulation through long-distance interaction, and are also one of the most well-known research objects in the field of three-dimensional genomics. They are mainly located in the open chromatin region (DNase I-sensitive part or ATAC-seq signal region), which can increase gene expression. In addition, the DNA sequence of the enhancer is rich in the binding elements of cell-specific transcription factors, and is rich in epigenetic regulatory factors such as transcription activator CBP/P300 and histone-modifying enzymes [110]. Enhancers are widely present in the whole genome and have strong spatiotemporal specificity. They can regulate the transcriptional regulation of target genes through long-distance interaction with target promoters [111]. Scientists have been interested in the study of enhancers for a long time, but due to the limitations of technology and scientific research, the study of enhancers has been stuck in the isolation and identification stage, lacking relevant theories and systematic research support. The development of three-dimensional genomics has injected a strong momentum into enhancer research. For example, Zhang et al.’s study revealed 40,000 long-range enhancer–promoter interactions in three different cell lines. Further analysis also showed that 40% of enhancers interacted with their neighboring genes, and the target genes of the remaining 60% enhancers were clearly identified [29].

In 2013, the Richard Young team proposed the concept of super enhancer [112]. On the basis of systematic analysis of enhancer data of different cells/developmental stages, they identified a class of important organisms in the process of life, including cell identity, cell fate, and growth cycle. The cluster of enhancers related to the learning process is named super-enhancer. Such elements can be identified in any cell, can define cell identity, are very sensitive to environmental changes, and have an important impact on the expression of proto-oncogenes [113, 114]. Some important traits and disease-related mutations are mostly located in the super-enhancer region, which can form the super-enhancer domain and express enhancer RNA [115, 116]. At present, there are many controversies about the concept of super-enhancers [117], but because it satisfies people’s needs for narrowing the research candidates, the research on super-enhancers has made explosive progress [118, 119]. For example, in cancer research, super-enhancers are used as a new type of drug target [120]; in cell research, they are used as candidates for nuclear microenvironment research [121]; in immune cells, they are used as key factors in developmental regulation [122, 123]. However, the specific composition, regulatory mechanism, and biological functions of super enhancers need to be further analyzed. There is no doubt that three-dimensional genomics technology is a powerful tool to analyze this series of problems, and its progress will lay the foundation for the study of accurately revealing the regulatory mechanism of super-enhancers.

## Precision biology and 3D genomics

The precision of precision biology is mainly reflected in the two aspects of resource conservation/maximization of effects and accurate targeting, and this must be based on an in-depth understanding of the underlying molecular mechanisms of existing biological processes/traits. The source of the concept of precision biology is quoted from precision medicine, and its foundation is based on the theory of big data, through the integration of genome, proteomics, and other omics technologies and biological processes, as well as the analysis, identification, verification, and application of biomarkers for large sample groups and specific trait types. The next step is to accurately find the target genes that determine the traits, and formulate personalized improvement strategies to accurately classify the dynamic spatiotemporal expression patterns of specific traits, and finally achieve the goal of improving and enhancing traits/biological individuals. In recent years, the scientific community has launched a series of precision biology projects. However, due to the lack of knowledge and understanding of basic gene functions and molecular mechanisms of action, the current progress tends to be slow [124, 125]. People have realized that big data should not only be used as the primary factor of precision biology; more attention should be paid to functional information, and the role of biological macromolecules in precision biology should be strengthened [126].

Three-dimensional genomics can reveal the general law of transcriptional regulation of a certain gene in the whole genome and, on this basis, further clarify the regulatory elements required in the transcription process of a specific gene, as well as the mutual cooperation relationship with other different genes, which is a single study of gene function, and molecular mechanism of action provides a brand-new approach [29]. Studies have shown that, when genes interact with different transcription elements/protein molecules, they can present different transcription patterns such as expression/closure. Further studies have shown that, when the concentration of transcription elements/protein molecules in the gene expression pattern changes, the level of gene expression also continuously increases or decreases [127]. The results of this study potentially indicate that the interactions between different genes/transcriptional elements in organisms/cells revealed by three-dimensional genomics can be used to determine the spatiotemporal expression patterns of genes under specific spatiotemporal conditions. The difference in their interaction mode and expression level will directly affect the level of expression of the gene or its temporal and spatial specificity. Therefore, the small changes in the expression pattern of the gene in the nucleus caused by the interaction of these genes/transcriptional elements can be directly used in the research of precision biology, and will directly promote the development of precision biology.

## Conclusions and prospects

In summary, through three-dimensional genomics, we can clearly understand the mechanism of spatial conformation of chromatin, the mechanism of gene transcription regulation, the formation mechanism of biological traits, the transmission mechanism of signal pathways, and the operating mechanism of the genome. The combination of 3C technology and other derivative sequencing technologies applied to three-dimensional genomics research has increasingly clarified the spatial conformation of the three-dimensional genomes of animals, plants, and microorganisms. Although related progress has been amazing in recent years, research on 4D nucleosomes is still at the tip of the iceberg [127]. Large improvements are still needed in experimental methods and calculation tools, and relevant technical standards have not yet been formally established. This has led to uneven data quality, and related hypotheses and theories still need to be improved. Because of the lack of common standards for the operation of testing experiments, the results of research cannot be directly compared. For example, the 5C technique allows a more comprehensive analysis of chromatin-loop interactions for a numerous genes by mapping interactions between multiple motifs in parallel, and is superior to 3C in statistical analysis, but is limited by the models and assumptions used to define the expected frequency of interactions [7]. Studies based on tomographic electron microscopy and combined labeling technology (ChromEMT) show that the three-dimensional structure of the nucleus shows that the accumulation of chromatin in the nucleus is based on the concentration of chromatin, which directly denies the chromatin classification proposed by the Hi-C technology structural hypothesis. It is believed that this aggregation of different concentrations determines the activity and recognition mechanism of DNA [128]. Nevertheless, the development of single-cell, high-throughput, and multi-omics 3D genomic analyses will allow us to understand in as much detail as possible the dynamic structure of the genomic organization under normal and diseased conditions. It will facilitate further exploration of gene expression regulation patterns, transcriptional–translational processes, and synergistic patterns of different genes.

There is a proliferation of emerging technologies, and the integration of emerging technologies with the 3D genome is becoming closer. Researchers developed CAPTURE technology based on proteomic and 3C interactions, as well as multi-ChIA technology based on single-molecule and single-cell principles [129]. At the same time, the ability of researchers to integrate different types of data (such as chromatin interaction data and imaging-based distance measurement) is still very limited, and there is a lack of methods that can measure and explain the differences in chromosomal and nuclear structure of different cells. The development of 3D genomics requires the establishment of a high-throughput technology-based gene function validation platform to verify data and different hypotheses, so as to establish research standards and technical platforms for 3D genomics.

The establishment of platforms and standards will help scientists to accumulate high-quality data more effectively and improve relevant theories, so that the development of 3D genomics will enter a virtuous cycle, while the development of 3D genomics will also provide a strong linkage and bridge for interdisciplinary research.

In addition, because of the large scale of data involved in the current research process of three-dimensional genomics, a series of different types of omics data are involved in the integration analysis. Commonly used visualization tools such as Genome Browser and DNA Database cannot well display the inherent characteristics and associations of 3D genomics data [130]. Therefore, a series of new visualization tools have been developed and used. These tools have their own emphasis and characteristics when displaying 3D genomics data, such as juicebox [131], 3D-genome Interaction Viewer and database [132], epigenome browser [133], and 3Disease browser [94]. Three-dimensional genome data show the interrelationships of different genome spatial conformations and different gene functions in multiple dimensions. However, the current tools cannot clarify the relationship among the three levels of interaction strength, spatial conformation, and gene synergy in 3D genome data. Especially at the user level, there are still major deficiencies in the usability and experience of terminal researchers; the newly developed three-dimensional genomics tools still have much room for improvement in terms of user experience and usability. The above shortcomings depend on further improvement in future work.

Finally, the development of precision biology depends on scientists’ perfection of a series of basic theories such as gene function, expression regulation mode, transcription and translation process, and synergy mode of different genes at the whole genome level. There is also a lack of a clear understanding of the molecular mechanism of the relationship between chromatin conformation and biological processes in the nucleus (including transcription, DNA replication, and chromosome separation). However, the development of three-dimensional genomics can provide a powerful bridge for the dynamic research of different biological processes. The gap between these basic biological phenomena and people’s cognition can be filled by means of high-level collaboration and multidisciplinary integration. This requires collaboration between research groups that specialize in imaging, genomics, computer science, and physics. However, the research of 3D genomics is still in its infancy, and how to transform the current obtained phased data into research results with clinical application value, and truly promote the development of related fields, is the top priority of 3D genomics research, such as gradually understanding the process of cancer and other diseases as well as chromatin internal and interchromatin structural changes. According to these changes, therapeutic targets can be found and targeted drugs developed, so as to regulate chromatin interactions for treating diseases. After the gene regulation network and mechanism of complex traits of animals and plants has been elucidated, how to use these advantages of three-dimensional structural characteristics to improve the level of biotechnology and improve the quality of life of people is a scientific question that needs to be explored by scientists. With the rapid development of single-cell, high-throughput, and multi-omics 3D genomic analysis, researchers are able to obtain deeper biological information to support the development of human society and the improvement of healthcare.

## Data Availability

Not applicable.

## Abbreviations

3D: Three-dimensional
HGP: Human Genome Project
ENCODE: Encyclopedia of DNA elements
3C: Chromatin conformation capture
4C: Circular chromatin conformation capture
5C: Chromatin conformation capture carbon copy
Hi-C: High-throughput chromosome conformation capture
ChromEMT: ChromEM tomography
GWAS: Genome-wide association study
FAANG: Functional Annotation of Animal Genomes
CT: Chromosome territory
CL: Chromatin loop
TADs: Topologically associated domains
mESC: Mouse embryonic stem cells
FISH: Fluorescence in situ hybridization
CHIP: Chromatin immunoprecipitation
LCR: Locus control regions
SPRITE: Split-pool recognition of interactions by tag extension
GAM: Genome architecture mapping
CAP-C: Chemical-crosslinking assisted proximity capture
Dam: DNA adenine methyltransferase
MINA: Multiplexed imaging of nucleome architectures
ORCA: Optical reconstruction of chromatin architecture
scHi-C: Single-cell Hi-C
CRISPR: Clustered regularly interspaced short palindromic repeats
EGFP: Enhanced green fluorescent protein
WGCNA: Weighted gene co-expression network analysis
